# A Case Management Program at Home to Reduce Fall Risk in Older Adults (the MAGIC Study): Protocol for a Single-Blind Randomized Controlled Trial

**DOI:** 10.2196/34796

**Published:** 2022-06-13

**Authors:** Silsam Napolitano Alberto, Juliana Hotta Ansai, Ana Luísa Janducci, João Vitor Businaro Florido, Areta Dames Cachapuz Novaes, Maria Joana Duarte Caetano, Paulo Giusti Rossi, Larissa Riani Costa Tavares, Stephen Ronald Lord, Karina Gramani-Say

**Affiliations:** 1 Department of Gerontology Federal University of São Carlos São Carlos Brazil; 2 Department of Physiotherapy Federal University of São Carlos São Carlos, SP Brazil; 3 Australia and School of Public Health & Community Medicine, University of New South Wales Sydney Australia

**Keywords:** accidental falls, risk management, aged, fall prevention

## Abstract

**Background:**

Individual case management programs may be particularly effective in reducing fall risk as they can better identify barriers and facilitators to health recommendations.

**Objective:**

This paper describes the protocol for a single-blind, parallel-group randomized controlled trial that aims to investigate the effectiveness and cost-effectiveness of a home-based multifactorial program targeting fall risk factors among people aged 60 years and over who have fallen at least twice in the past 12 months (the MAGIC trial).

**Methods:**

Older people with a history of at least 2 falls in the last year will be divided into 2 groups. The intervention group will receive case management at home for reducing the risk of falls, including a multidimensional assessment, explanation of fall risk factors, and elaboration and monitoring of an individualized intervention plan based on the identified fall risk factors, personal preferences, and available resources. The control group will be monitored once a month. Assessments (clinical data, fall risk awareness, physical and mental factors, safety at home, feet and shoes, and risk and rate of falls) will be carried out at baseline, after 16 weeks of the intervention, and at the posttrial 6-week and 1-year follow-up. After 16 weeks of the intervention, satisfaction and adherence to the intervention will also be assessed. Economic health will be evaluated for the period up to the posttrial 1-year follow-up.

**Results:**

Data collection started in April 2021, and we expected to end recruitment in December 2021. This case management program will address multifactorial assessments using validated tools and the implementation of individualized intervention plans focused on reducing fall risk factors.

**Conclusions:**

This trial may provide reliable and valuable information about the effectiveness of case management for increasing fall risk awareness and reducing fall risk in older people.

**Trial Registration:**

Brazilian Clinical Trials Registry (ReBec) RBR-3t85fd; https://ensaiosclinicos.gov.br/rg/RBR-3t85fd

**International Registered Report Identifier (IRRID):**

DERR1-10.2196/34796

## Introduction

One of the challenges of aging is the social and health costs arising from falls—a major cause of disability and death among older adults. The economic impact of falls affects family, community, and society [1]. Between 2011 and 2015, 43,400 fall-related deaths and 240,000 fall-related hospitalizations occurred among older Brazilian people [2,3]. In general, falls result from multiple risk factors. Reducing risk factors for falls through an intervention based on case management may be particularly beneficial for preventing falls in older adults [4].

Case management interventions allow professionals to develop and implement individualized plans for preventing falls and consequences [5,6]. The case manager ensures follow-up of the plans in accordance with the older adult and family, identifies barriers to their implementation, and motivates the older person to adhere to the program [5]. Moreover, in a cross-sectional study, the preference of the older people in relation to different formats of programs for falls was verified. Older people with a greater concern about falls and disposition to participate in some program prefer participating in personalized and home-delivered programs based on their individual characteristics, difficulties, and limitations when compared to programs conducted in groups [7].

A Cochrane systematic review has shown that integrated multifactorial assessments and individualized interventions are effective in reducing the rate of falls [8,9]. Although these studies provide good evidence for fall prevention, there is a lack of trials investigating multifactorial interventions based on individualized fall risk factors, such as case management interventions. In addition, there is a need for studies that assess acceptance and adherence to multifactorial programs for reducing fall risk [8,9], taking into consideration monthly falls monitoring and the presence of managers within the work team.

There is a scarcity of randomized controlled trials involving case management for reducing fall risk. Individual case management programs may be particularly effective in reducing fall risk, as they can better identify barriers and facilitators to health recommendations. This model of care can offer physical, psychological, and social assistance that goes beyond the traditional models of health care for fallers.

Considering the aforementioned discourse, we designed a home-based multifactorial program targeting fall risk factors among people aged 60 years and over who have had a fall at least twice in the past 12 months. The proposed intervention program consists of individualized management of fall risk factors. This study protocol details the MAGIC trial, outlining the design of the study, describing their objectives, methodology, and overall organization of the research to be carried out.

## Methods

### Trial Design and Setting

A parallel-group, single-blind randomized controlled trial with a block randomized design (allocation ratio 1:1) will be carried out. The trial will be performed in São Carlos, Brazil, and surrounding cities, where 16% of the local population is aged 60 years and over (Multimedia Appendix 1).

### Study Population

Participants will be recruited through health and social services, open registration via telephone, and a volunteer database of the research group. The study will be promoted via leaflets, posters, social networks, and local radio and television.

Eligible people will be community older adults aged 60 years and over, be residents of São Carlos and surrounding regions, be noninstitutionalized, and have a history of falls in the last year. Inclusion criteria will be having a history of at least 2 falls in the last year, being willing to participate in the interventions and assessments, being able to walk with or without walking devices, and being available to be contacted by telephone. Exclusion criteria will be having severe and uncorrected visual or auditory disorders that affect communication during assessment and intervention, having active inflammatory and neurological diseases that severely interfere with balance performance (advanced Parkinson disease—stage 5 on the modified Hoehn and Yahr Scale and not being regular users of antiparkinsonian medications, multiple sclerosis, Huntington disease, dementia, uncontrolled vestibulopathy, epilepsy, traumatic brain injury, and severe motor sequelae of stroke), or participating in regular and systematic physical activity for a total duration of ≥150 minutes per week.

### Randomization and Blinding

A randomization sequence will be created by an external researcher with Random Allocation software (Windows Inc) with a 1:1 allocation using random block sizes. The allocation sequence will be concealed from the external researcher enrolling participants in sequentially numbered, opaque, sealed, and stapled envelopes. Corresponding envelopes will be opened after assessment at baseline by another independent researcher, who will be responsible for advising participants of their allocation by telephone.

Assessors will be blinded to randomization until the end of the study. Owing to the nature of the trial, the researchers responsible for the intervention and the volunteers will not be blinded to randomization.

### Intervention

The intervention protocol follows all protective measures recommended in response to the COVID-19 pandemic. The intervention group (IG) will receive a remote case management intervention at home over 16 weeks, once a week by video or a telephone call. Volunteers who have regular access to the internet and a smartphone or computer made weekly calls through video, while those with limited access were contacted by telephone. The control group (CG) will be followed by monthly telephone calls. The intervention will be carried out by 2 trained researchers, scheduled to start in April 2021.

Case managers are responsible for monitoring the volunteers and their families among the intervention proposals to reduce each of the modifiable fall risk factors in the initial assessment. These managers are trained gerontologists who, in addition to having pre-established proposals, hold discussions fortnightly to discuss cases with the entire intervention team, ensuring the best guidelines for volunteers and the alignment of manager´s actions. According to the Brazilian Society of Geriatrics and Gerontology, gerontologists have training in different areas of knowledge (psychology, social work, nutrition, occupational therapy, law, etc), so they are able to plan, coordinate, and evaluate health actions related to older people; in addition, they can deliver biopsychosocial care.

In the first week, for the IG, a multidimensional fall risk assessment will be applied (Multimedia Appendix 2) [10]. In the second session, the case managers will discuss and explain the identified fall risk factors to the participant and his or her caregiver. An individualized intervention proposal based on the identified fall risk factors will be devised considering participants’ preferences and priorities. All IG volunteers will be encouraged to carry out a home-based multicomponent physical exercise program delivered through recorded videos, guidelines, or printed booklets. The exercise program will consist of trunk and lower limb muscle–strengthening, aerobic conditioning, gait, and balance and stretching exercises at moderate intensity, performed twice per week (30-60 minutes per session) with individualized progression every 2 weeks. The exercise program is based on a previous protocol [11] and in line with the recommendations of the American College of Sports Medicine [12], having been adapted for community older adults in the primary health context. Volunteers who agree to participate in the intervention with physical exercises will have to present a medical certificate before carrying out the activities.

In the third session, an individualized intervention plan will be developed with the volunteer and his or her caregiver, taking into consideration priority of the identified fall risk factors, personal preferences, and available resources. During the 16 weeks of the intervention, the case manager, the volunteer, and his or her caregiver will implement the previously agreed plan, which includes recommendations and guidelines, dialogue with suppliers, and referrals for specialized programs. If needed, the plan will be monitored and reviewed by weekly telephone or video calls. During the monitoring, the manager will check if the participant has any difficulty in carrying out the requested activities and will identify what assistance should be offered and what possible changes are needed [5,6]. All cases will be discussed with case managers and 2 specialists in the field. All recommendations, in case of resistance, will be facilitated for better adhesion based on support materials and personal preferences. If there is still refusal, awareness and maintenance of daily risk behaviors will be prioritized so that no accidents happen.

**Figure 1:**
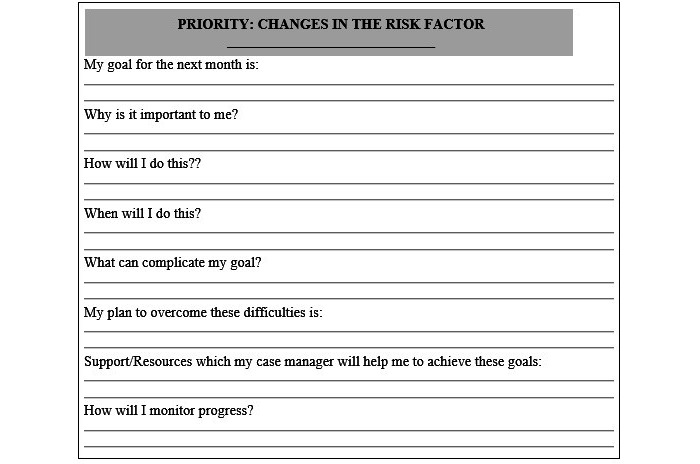
Recommendations for reducing fall risk.

After 16 weeks of the intervention, all volunteers will undergo 6 weeks of detraining (follow-up) in which they will be asked to carry out their usual activities. At the end of the study, all volunteers will receive a booklet of recommendations for reducing fall risk (Figure 1). If positive results are identified, older adults of the CG will be invited to undertake the intervention.

### Outcome Measures

#### Overview

Assessments will be conducted at participants’ homes at baseline, after 16 weeks of the intervention, and at 6 weeks and 1 year post trial completion. The volunteers will be instructed to wear comfortable clothing, closed shoes, not to perform vigorous exercises the day before the assessment, and to wear hearing or visual aids if needed.

Baseline assessments include sociodemographic measures (age, sex, race, marital status, years of schooling, living alone, and income) and general health (BMI; medication use; morbidities; foot problems including dryness of skin, calluses, fissures, open ulcers, and deformities; history of falls, hospitalizations, level of physical activity measured with the Modified Baecke Questionnaire for the Elderly; general health assessed with the Self-Rated Health questionnaire; functional activities evaluated with the Lawton-Brody Scale; and risk of fracture measured with the Fracture Risk Assessment Tool—FRAX).

Primary and secondary outcomes will be reassessed after 16 weeks of the intervention and at the posttrial 6-week follow-up. In addition, after 16 weeks of the intervention, satisfaction and adherence to the intervention will also be assessed among the IG participants. Prospective falls will be collected from baseline to 1-year follow-up. The economic health assessment will be carried out for the period up to the posttrial 1-year follow-up.

#### Primary Outcome Measure

The primary outcome is fall risk awareness and behavior change, which will be assessed using the Falls Risk Awareness Questionnaire (Brazil). A score range from 0 to 32 points and higher scores indicate greater knowledge about fall risk factors [13].

#### Secondary Outcome Measures

Secondary outcomes are falls, physical function (muscle strength of lower limbs, balance, and mobility), mental functions (cognition, depressive symptoms, and fear of falling), fall risk, safety at home, feet and footwear, satisfaction with and adherence to the intervention, and health care costs.

Prospective falls will be collected from baseline to 1-year follow-up using falls calendars and monthly telephone calls. Muscular strength of hip, knee, and ankle joints will be evaluated using the Lafayette dynamometer. In addition, muscle strength, mobility, and balance will be assessed with the Brazilian version of the Short Physical Performance Battery (SPPB). The SPPB consists of 3 tests: Balance Test, Gait Speed Test, and Chair Stand Test [14]. The scores range from 0 to 4, and the total ranges from 0 to 12 points, with higher scores indicating better lower limb function [14]. Balance will also be assessed with the MiniBest test, which has a total score of 28 points, and higher scores indicate better balance [15].

Cognition will be assessed with Addenbrooke’s Cognitive Examination-Revised screening battery, which has a maximum score of 100 points, with a score of ≤78 points indicating cognitive impairment [16]. Digit Span Forward will be used to assess attention and immediate memory deficits (indicated by scores less than 6 points) and Digit Span Backward for measuring attention and working memory deficits (indicated by scores less than 4 points) [17]. To assess depressive symptoms, the Geriatric Depression Scale will be used. Higher scores on this 15-point scale indicate a greater risk of depressive symptoms. The Falls Efficacy Scale-International will be used to assess fear of falling [18]. A total score of ≥23 points is associated with occasional falls, and a score of >31 is associated with recurrent falls [18].

Fall risk will be assessed with the Falls Risk Assessment Tool. The score varies from 0 to 5 points, with a score of 3 or more points indicating a high risk of falls [19]. Regarding home safety, the Home Falls and Accidents Screening Tool will be used. The maximum score is 25 points, and higher scores are indicative of a greater risk of falls and home accidents [20].

For feet and footwear, the Manchester Foot Pain and Disability Index will be used. A total score of ≥2 points indicate disability associated with foot pain [21]. The tactile sensitivity of feet will be assessed using the 10-gram Semmes-Weinstein monofilament on hallux, the second and fifth metatarsals, 3 points of the forefoot, and 1 point of the midfoot and heel. Flexibility (dorsiflexion and plantar flexion) will be assessed by a goniometer. The characteristics of footwear that the volunteers usually wear at home and when they get up at night will be documented.

After 16 weeks, adherence to the case management program will be assessed by diary entries. Adherence of ≥70% to recommendations will be considered satisfactory [22,23]. In addition, questionnaires will be used to assess reasons for adhering or not adhering to treatment. The prevalence of each recommendation offered in the case management and adherence to these recommendations will also be assessed, using a 3-point scale (total adherence, partial adherence, and nonadherence) [24]. To assess satisfaction of the intervention after 16 weeks, a questionnaire based on the Short Assessment of Patient Satisfaction will be used [25]. A question will be added about the satisfaction of overall care (“How would you rate the health care you received in the past 16 weeks?”), with scores ranging from 0=worst possible care to 10=best possible care.

For economic analyses, the incremental cost effectiveness ratio will be calculated as the difference between the total cost of the IG and that of the CG. Furthermore, the use of health services will be analyzed. The EuroQol-5D is used in many cost-effectiveness studies, which allows the comparison of effects generated by interventions for any disease. Its score ranges from 1 to 3 for each item, allowing 243 different health profiles [26,27].

### Power and Sample Size

For the primary outcome (fall risk awareness), the sample size was calculated using the G*Power 3.1 software, based on type of study design (2-way ANOVA), type I error of 5%, statistical power of 80%, moderate effect size (0.20), and number of groups (n=2). This revealed that a total sample size of 42 is required. Allowing for 20% dropout, we will recruit a sample size of 60 people.

### Statistical Analysis

For statistical analysis, a significance level of 5% will be adopted, and SPSS (version 22.0; IBM Corp) will be used. The analysis will be performed by intention to treat. The Kolmogorov-Smirnov normality test will be applied for all continuous variables to verify data distribution. To compare groups with regard to clinical and sociodemographic characteristics, the chi-square association test and the independent samples *t* test will be used. To test the interaction between groups and assessments, a 2-way, repeated measures ANOVA will be used. If an interaction is identified, simple main effects analyses will be performed, with adjustment for multiple comparisons (Bonferroni correction). To verify which factors influence adherence to the intervention (frequency of ≥70%), a univariate logistic regression analysis will be used. To check which factors influence satisfaction with the intervention, a simple linear regression analysis will be applied.

### Ethics Approval, Monitoring, and Dissemination

**Figure 2:**
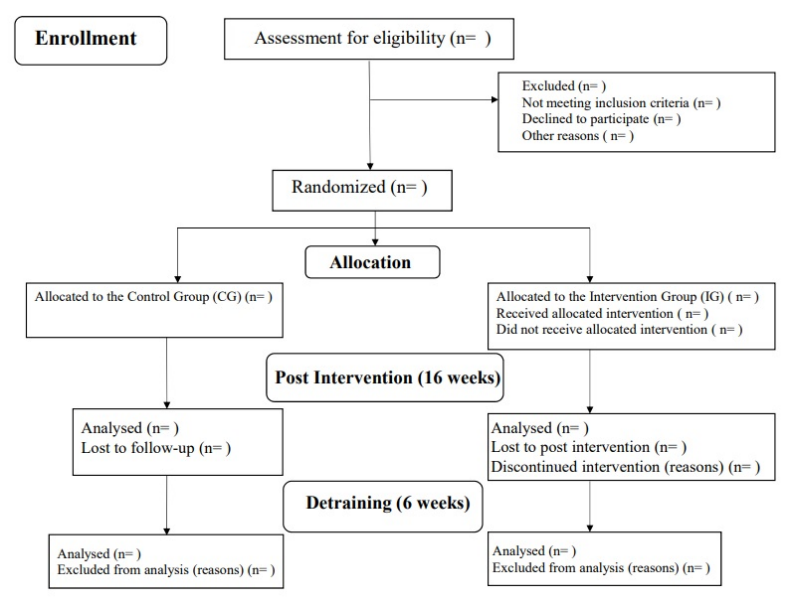
CONSORT flowchart 2010.

The trial was approved by the research ethics committee of Federal University of São Carlos (CAAE: 34350620.7.0000.5504). Regarding monitoring, the researchers will be responsible for making decisions related to any need for changes to the assessment and intervention in the presence of adverse effects during the research. Data will be used only for scientific purposes with the utmost confidentiality and will not be transferred to any person or entity outside of the research team. Participant data will not be released. There will be no personal expenses or any benefits for the participant. If there is any damage resulting from the research, compensation will be guaranteed. The participant will have the right to withdraw from the study at any time, if they will, without prejudice to them. If necessary, at all stages of the study, participants will have access to the researchers for further enquiries (Figure 2).

## Results

This is a protocol for a single-blind, parallel-group randomized controlled trial funded in July 2021 by Conselho Nacional de Desenvolvimento Científico e Tecnológico (CNPq), Fundação de Amparo à Pesquisa do Estado de São Paulo (FAPESP), and Coordenação de Aperfeiçoamento de Pessoal de Nível Superior (CAPES; grant code 001), Approved by Registro Brasileiro de Ensaios Clínicos (ReBEC; RBR-3t85fd), with data collection started in April 2021 and expected to end in December 2021. The number of recruited volunteers as of submission of this manuscript is 52.

## Discussion

The MAGIC trial aims to provide rigorous direct evidence about the effectiveness and cost-effectiveness of a home-based multifactorial program targeting fall risk factors among people aged 60 years and over who have fallen at least twice in the past 12 months. We hypothesize that the IG presents better awareness of fall risk and positive behavior change in reducing fall risk factors identified by case management, thus decreasing new episodes of falls with an increasing volume of physical activity after the 16-week intervention compared to the CG; in addition, we expect that community older people in the IG will continue to show the positive results obtained after the 6-week follow-up compared to the CG.

The strengths of this MAGIC study are the follow-up of individualized case management that offers greater attention to the older people in the home environment and includes the family or caregiver in the action plan for behavior change in order to reduce the identified risk factors for falls. In addition, the program offers monitoring of the older people by a physical therapist to implement a protocol for safe physical activities at home.

The researchers may encounter some limitations during this randomized controlled trial, including difficulties in recruitment, restrictions on home visits owing to the COVID-19 pandemic, and poor compliance with the recommendations proposed by the case managers. If needed, potential solutions will be the use of remote assessments by video and telephone calls and the inclusion of additional recruitment sites.

The findings of this study may provide reliable and valuable information about the effectiveness of case management for increasing fall risk awareness and reducing fall risk in older people. This study protocol detailed the design and methodology of the research to support the transparency of results. This may also assist the design of future studies.

## Appendix

Multimedia Appendix 1 Table 1: Description of Study Period.

Multimedia Appendix 2 Table 2. Intervention plan sheet, including modified STRIDE Intervention risk factors and priorities [12].

Multimedia Appendix 3 Peer Review Reports from the Auxílio à Pesquisa Regular - Fundação de Amparo à Pesquisa do Estado de São Paulo (FAPESP), The São Paulo Research Foundation (Brazil).

## Abbreviations

CAPES: Coordenação de Aperfeiçoamento de Pessoal de Nível Superior
CG: control group
CNPq: Conselho Nacional de Desenvolvimento Científico e Tecnológico
FAPESP: Fundação de Amparo à Pesquisa do Estado de São Paulo
IG: intervention group
ReBEC: Registro Brasileiro de Ensaios Clínicos
SPPB: Short Physical Performance Battery
