# Supplementary material for: A Case Management Program at Home to Reduce Fall Risk in Older Adults (the MAGIC Study): Protocol for a Single-Blind Randomized Controlled Trial
Source: JMIR Res Protoc. 2022 Jun 13;11(6):e34796. doi: 10.2196/34796 (PMC9237774; doi:10.2196/34796)
Supplement: Multimedia Appendix 1 [file resprot_v11i6e34796_app1.docx]

**Table 1:** Description of Study Period.

|  | **STUDY PERIOD** | | | | | |
| --- | --- | --- | --- | --- | --- | --- |
|  | Enrolment | Allocation | Post Allocation | | | Close-out |
| **TIMEPOINT** | **-t_1_ (BEFORE BASELINE)** | **0 (BASEFORE BASELINE)** | **t_1_ (BASELINE)** | **t_2_**  **(AFTER 16 WEEKS OF TRAINING)** | **t_3_**  **(6 WEEKS OF DETRAINING)** | **T_3_ (AFTER 12 MONTHS OF BASELINE)** |
| **ENROLMENT:** |  |  |  |  |  |  |
| Eligibility screen | X |  |  |  |  |  |
| Informed consent | X |  | X |  |  |  |
| Pilot tests of evaluation and intervention | X |  |  |  |  |  |
| Allocation |  | X |  |  |  |  |
| **INTERVENTIONS:** |  |  |  |  |  |  |
| [Intervention Group] |  |  | X | X | X | X |
| [Control Goup] |  |  | X | X | X | X |
| **ASSESSMENTS:** |  |  |  |  |  |  |
| **Inicial Evaluation** (sociodemografic characteristics and general health |  |  | X |  |  |  |
| **Motor measurements:** SPPB, Lafayette dynamometer, MNSI-BRASIL. **Neuropsycological measurements:** GDS, FES-I, ACER-R, Digit Span. **Home security measure:** HOMEFAST |  |  | X | X | X | X |
| **Health economic evaluation:** ICER, EuroQol-5D |  |  |  |  |  | X |
| **Adherence and satisfaction to the intervention:** adherence questionnaire, SAPS, satisfaction questionnaire. |  |  |  | X |  |  |
| **Falls:** calendar of falls, monthly phone calls |  |  | X | X | X | X |
