# Supplementary material for: A Case Management Program at Home to Reduce Fall Risk in Older Adults (the MAGIC Study): Protocol for a Single-Blind Randomized Controlled Trial
Source: JMIR Res Protoc. 2022 Jun 13;11(6):e34796. doi: 10.2196/34796 (PMC9237774; doi:10.2196/34796)
Supplement: Multimedia Appendix 2 [file resprot_v11i6e34796_app2.docx]

**Table 2.** Intervention plan sheet including risk factors and priorities based on the STRIDE Intervention^14^

| MY FALLS RISK ASSESSMENT | | | |
| --- | --- | --- | --- |
| Participant Name: ____________________________________________________________________________  Date: __/__/__ …………………… Responsible manager:__________________ Partner:___________________ | | | |
| **Risk Factor** | **Why Does It Matter?** | **Is this a risk for me?** | **Is this a priority for me?** |
| History of falls | Older adults who suffer a fall are more likely to fall again. The risk of falling increases with the number of risk factors. | Yes ( )  No ( ) | Yes ( )  No ( ) |
| **Comments:____________________________________________________________________________________________________________________________________________________________________________** | | | |
| Medications | Drugs that cause dizziness or tiredness can increase chances of falling. | Yes ( )  No ( ) | Yes ( )  No ( ) |
| **Comments:____________________________________________________________________________________________________________________________________________________________________________** | | | |
| Vitamin D supplements | People who do not take vitamin D supplements are more likely to fall and suffer an injury. | Yes ( )  No ( ) | Yes ( )  No ( ) |
| **Comments:____________________________________________________________________________________________________________________________________________________________________________** | | | |
| Changes in leg strength, balance and/or walking | People with decreased leg strength and changes in balance and /or mobility are more likely to trip, slip and fall. | Yes ( )  No ( ) | Yes ( )  No ( ) |
| **Comments:____________________________________________________________________________________________________________________________________________________________________________** | | | |
| Vision problems | Vision problems can lead to mistakes when walking. | Yes ( )  No ( ) | Yes ( )  No ( ) |
| **Comments:____________________________________________________________________________________________________________________________________________________________________________** | | | |
| Diseases like osteoporosis, urinary incontinence, heart disease, dizziness or cerebellar dysfunction | Such diseases can predispose falls or bring consequences such as difficulties to perform daily activities and increase chance of falling. | Yes ( )  No ( ) | Yes ( )  No ( ) |
| **Comments:____________________________________________________________________________________________________________________________________________________________________________** | | | |
| Heart rate | Changes in heart rate can cause shortness of breath, dizziness and fainting and increase chance of falling. | Yes ( )  No ( ) | Yes ( )  No ( ) |
| **Comments:____________________________________________________________________________________________________________________________________________________________________________** | | | |
| Feet and Footwear | Problems with feet and shoes can make walking difficult and increase chance of falling. | Yes ( )  No ( ) | Yes ( )  No ( ) |
| **Comments:____________________________________________________________________________________________________________________________________________________________________________** | | | |
| Low pressure (postural hypotension) | Low blood pressure when a person changes position can increases chance of falling. | Yes ( )  No ( ) | Yes ( )  No ( ) |
| **Comments:____________________________________________________________________________________________________________________________________________________________________________** | | | |
| Safety at home | Objects on the floor, loose carpets, poor lighting and handrails can increase the chances of tripping, slipping and falling. | Yes ( )  No ( ) | Yes ( )  No ( ) |
| **Comments:____________________________________________________________________________________________________________________________________________________________________________** | | | |
| Functional deficit and use of walking device (cane, walker) | Walking device with inadequate height or with worn and loose tips can increase chance of falling. | Yes ( )  No ( ) | Yes ( )  No ( ) |
| **Comments:____________________________________________________________________________________________________________________________________________________________________________** | | | |
| Fear of falling | Failure to perform activities due to fear of a new fall over time can result weakness and lack of balance and increase chance of falling. | Yes ( )  No ( ) | Yes ( )  No ( ) |
| **Comments:____________________________________________________________________________________________________________________________________________________________________________** | | | |
| Living alone | Older adults who need care are more likely to fall. | Yes ( )  No ( ) | Yes ( )  No ( ) |
| **Comments:____________________________________________________________________________________________________________________________________________________________________________** | | | |
| Pain | Presence of pain, especially in knees and hips, can damage posture, mobility, strength and balance and increase chance of falling. | Yes ( )  No ( ) | Yes ( )  No ( ) |
| **Comments:____________________________________________________________________________________________________________________________________________________________________________** | | | |
| Depression | Older adults who feel very sad tend to become more closed, isolated, inattentive and with bent posture, which increase chance of falling. | Yes ( )  No ( ) | Yes ( )  No ( ) |
| **Comments:____________________________________________________________________________________________________________________________________________________________________________** | | | |
