# Supplementary material for: A Case Management Program at Home to Reduce Fall Risk in Older Adults (the MAGIC Study): Protocol for a Single-Blind Randomized Controlled Trial
Source: JMIR Res Protoc. 2022 Jun 13;11(6):e34796. doi: 10.2196/34796 (PMC9237774; doi:10.2196/34796)
Supplement: Multimedia Appendix 3 [file resprot_v11i6e34796_app3.pdf]

VISUALIZAÇÃO DE DESPACHO

**Processo**	2021/00181-1
**Linha de Fomento**	Programas Regulares / Auxílios a Pesquisa / Projeto de Pesquisa / Projeto de Pesquisa - Regular - Fluxo Contínuo
**Situação**	Em Contratação
**Vigência**	01/02/2022 a 31/01/2024
**Beneficiário**	Juliana Hotta Ansai
**Responsável**	Juliana Hotta Ansai
**Vínculo Institucional do Processo**	Centro de Ciências Biológicas e da Saúde/CCBS/UFSCAR
**Título**	Efetividade de uma intervenção baseada em gestão de casos sobre fatores de risco neuropsicológicos de quedas e sua aderência e satisfação de idosos caidores da comunidade: um ensaio clínico randomizado

Folha de Despacho para Reconsideração 001 - Projeto de Pesquisa - Regular

Resultado

Concedido

Datas do Despacho

**Emitido em :**	29/11/2021

Orçamento Consolidado

Benefícios	Solicitado		Despacho	
	Valor (R$)	Valor (US$)	Valor (R$)	Valor (US$)
Capital				
Material Permanente	9.979,30	0,00	6.846,00	0,00
Custeio				
Despesas de Transporte	6.999,00	0,00	6.999,00	0,00
Diárias	2.660,00	0,00	2.660,00	0,00
Material de Consumo	2.319,99	0,00	5.116,43	0,00
Serviços de Terceiros	500,00	850,00	500,00	850,00
Reserva Técnica - Benefícios Complementares	16.000,00	0,00	32.000,00	0,00
Reserva Técnica - Custo de Infraestrutura Direta do Projeto	4.050,86	0,00	4.051,33	0,00
Provisão para Importação	0,00	127,50	0,00	127,50
Total	42.509,15	977,50	58.172,76	977,50

Quotas de Bolsa

Solicitado				Despacho			
Modalidade / Nível	Carga Horária	Duração (Meses)	Quantidade	Modalidade / Nível	Carga Horária	Duração (Meses)	Quantidade
TT-3	20	24	1	TT-3	20	24	1

Equipe de Projeto

Membros da Equipe - Solicitados

Nome	Função	BC Solicitado	Período Solicitado
Juliana Hotta Ansai	Pesquisador Responsável	Sim	01/08/2021 - 31/07/2023
Karina Gramani Say	Pesquisador Associado	-	01/08/2021 - 31/07/2023
Larissa Riani Costa Tavares	Pesquisador Associado	-	01/08/2021 - 31/07/2023
Maria Joana Duarte Caetano	Pesquisador Associado	-	01/08/2021 - 31/07/2023
Paulo Giusti Rossi	Pesquisador Associado	-	01/08/2021 - 31/07/2023
Stephen Ronald Lord	Pesquisador Associado	-	01/08/2021 - 31/07/2023
Ana Luísa Janducci	Estudante sem Bolsa	-	01/08/2021 - 31/07/2023
Areta Dames Cachapuz Novaes	Estudante sem Bolsa	-	01/08/2021 - 31/07/2023
DANIELA LEMES FERREIRA	Estudante sem Bolsa	-	01/08/2021 - 31/07/2023
Dayane Melo Campos	Estudante sem Bolsa	-	01/08/2021 - 31/07/2023
José Emanuel Alves	Estudante sem Bolsa	-	01/08/2021 - 31/07/2023
João Vitor Businaro Florido	Estudante sem Bolsa	-	01/08/2021 - 31/07/2023
Maria Juana Beatriz Lima Candanedo	Estudante sem Bolsa	-	01/08/2021 - 31/07/2023
Renata Carolina Gerassi	Estudante sem Bolsa	-	01/08/2021 - 31/07/2023
SILSAM NAPOLITANO ALBERTO	Estudante sem Bolsa	-	01/08/2021 - 31/07/2023

Membros da Equipe - Despachados

Nome	Função	BC Despachado	Período Despachado
Juliana Hotta Ansai	Pesquisador Responsável	Sim	01/02/2022 - 31/01/2024
Karina Gramani Say	Pesquisador Associado	-	01/02/2022 - 31/01/2024
Larissa Riani Costa Tavares	Pesquisador Associado	-	01/02/2022 - 31/01/2024
Maria Joana Duarte Caetano	Pesquisador Associado	-	01/02/2022 - 31/01/2024
Paulo Giusti Rossi	Pesquisador Associado	-	01/02/2022 - 31/01/2024
Stephen Ronald Lord	Pesquisador Associado	-	01/02/2022 - 31/01/2024
Ana Luísa Janducci	Estudante sem Bolsa	-	01/02/2022 - 31/01/2024
Areta Dames Cachapuz Novaes	Estudante sem Bolsa	-	01/02/2022 - 31/01/2024
DANIELA LEMES FERREIRA	Estudante sem Bolsa	-	01/02/2022 - 31/01/2024
Dayane Melo Campos	Estudante sem Bolsa	-	01/02/2022 - 31/01/2024
José Emanuel Alves	Estudante sem Bolsa	-	01/02/2022 - 31/01/2024
João Vitor Businaro Florido	Estudante sem Bolsa	-	01/02/2022 - 31/01/2024
Maria Juana Beatriz Lima Candanedo	Estudante sem Bolsa	-	01/02/2022 - 31/01/2024
Renata Carolina Gerassi	Estudante sem Bolsa	-	01/02/2022 - 31/01/2024
SILSAM NAPOLITANO ALBERTO	Estudante sem Bolsa	-	01/02/2022 - 31/01/2024

Dados de Execução

**Data Início**	01/02/2022
**Duração**	24 mês(es)
**Data Término**	31/01/2024
**Área de alocação de recursos**	Saúde
**Relatório Científico (Quantidade)**	2
**Relatório Científico (Datas)**	30/01/2023<br>28/02/2024
**Prestação de Contas (Quantidade)**	2
**Prestação de Contas (Datas)**	28/02/2023<br>28/02/2024
**Categoria de Pesquisa**	T/PP

Observações / Transcrições / Frases

Observações ao Responsável

Comunicamos que sua solicitação de auxílio à pesquisa, constante do processo acima referido, foi analisada pela assessoria da FAPESP, tendo sido aprovada.

Cabe-nos informar que alguns dos itens orçamentários solicitados podem não ter sido aprovados, ou aprovados com valores inferiores. Por favor, aguarde o email com as instruções para confirmação de interesse pela concessão.

A utilização da concessão deve seguir o Manual de Instruções para Uso dos Recursos e Prestação de Contas de Auxílios e de Reserva Técnica.

Para conhecimento do conteúdo do despacho, por favor, acesse o Sistema SAGe (www.fapesp.br/sage), selecionando o item do menu Meus Processos>>Número do Processo e, em Mais Informações, a opção Despacho.

Por favor, para qualquer consulta ou comunicação sobre esta correspondência, use exclusivamente os serviços do "Converse com a FAPESP" em www.fapesp.br/converse.

Atenciosamente,

Luiz Eugênio A. M. Mello
Diretor Científico

OBSERVAÇÃO:
1. Foi concedida cota de bolsa orçamentária, conforme discriminado no Termo de Outorga, podendo haver dedução de bolsa anterior quando a regra se aplicar.
O candidato selecionado somente poderá iniciar suas atividades no projeto após a aprovação de seu cadastro, que deve ser submetido via SAGe, conforme Instrução Normativa específica para cada modalidade, constante do site da FAPESP em www.fapesp.br/bolsas/bolsasconcedidasemauxilios. Não serão feitos pagamentos ou reembolsos referentes a bolsistas fora do respectivo processo SAGe.
Possíveis renovações da referida cota de bolsa deverão ser solicitadas no Auxilio, por meio de solicitação de alteração da concessão inicial, no momento da entrega do Relatório Científico. A vigência da bolsa não poderá ultrapassar a vigência do auxílio.

Frases para o Responsável

Não há frases associadas.

Transcrição de Parecer para o Responsável

ANÁLISE DA PROPOSTA. A análise de propostas submetidas na modalidade Auxílio à Pesquisa Regular compreende a Avaliação do Projeto de Pesquisa, do Histórico Acadêmico do Pesquisador Responsável pelo Projeto e do Orçamento. Este formulário é composto por três seções referentes a cada uma das partes, tendo ao final uma Apreciação Geral do Projeto de Pesquisa (AGP).

I. Sobre o PROJETO DE PESQUISA proposto

Critério 1: Projeto de pesquisa original, competitivo internacionalmente e bem apresentado. A proposta em análise atende ao critério:

[ ] Muito [X] Bastante [ ] Razoavelmente [ ] Pouco [ ] Nada

Por favor, justifique a escolha acima (A força da justificativa será fator decisivo na análise da proposta pela FAPESP):

Houve melhora substancial no projeto de pesquisa com as reformulações realizadas. A contextualização do estudo está mais robusta. Do ponto de vista do método há ainda a meu ver uma questão a ser resolvida que é o fato do exercício não ser potencialmente entregue a todos os participantes, uma vez que será facultativo ao idoso/familiar cuidador aceitar ou não a intervenção. Isso criará dois subgrupos de intervenção, sendo que um deles terá maior possibilidade de efetividade sobre os desfechos primários, tendo em vista os efeitos conhecidos do exercício sobre as variáveis neuropsicológicas. Eu sugiro que o exercício seja entregue a todos os participantes e sua adesão mensurada. Caso os autores queiram justificar que o exercício deva ser facultativo, sugiro o planejamento de uma análise de subgrupo e, neste caso o tamanho da amostra deverá ser ampliado. Considero que um follow-up de 6 semanas seja de curto prazo. Efeitos de longo prazo com reavaliação em 12 semanas e 24 semanas seriam particularmente interessantes para saber se possíveis benefícios seriam mantidos ao longo do tempo. Embora a redução de quedas não seja um desfecho primário, todo o arcabouço do estudo se baseia em fatores de risco modificáveis para prevenção de quedas. Neste sentido o acompanhamento do número das quedas durante o seguimento é recomendável, por meio de telefonemas e diários de quedas.

Critério 2: Os desafios de pesquisa estão adequadamente formulados e situados frente ao estado da arte e à literatura existente. A proposta em análise atende ao critério:

[ ] Muito [X] Bastante [ ] Razoavelmente [ ] Pouco [ ] Nada

Por favor, justifique a escolha acima (A força da justificativa será fator decisivo na análise da proposta pela FAPESP):

Sim os desafios estão adequadamente justificados. A integração de serviços e intervenções centrados nas necessidades dos idosos e seus familiares é um desafio dos sistemas de saúde que enfrentam o aumento da população idosa e da complexidade de cuidados.

Critério 3: A metodologia é adequada e está bem justificada e suficientemente detalhada. A proposta em análise atende ao critério:

[ ] Muito [X] Bastante [ ] Razoavelmente [ ] Pouco [ ] Nada

Por favor, justifique a escolha acima (A força da justificativa será fator decisivo na análise da proposta pela FAPESP):

Os métodos foram aprimorados em termos das descrições das intervenções e desfechos. A entrega das intervenções usando plataformas digitais é uma proposta que cabe bem ao momento em que estamos vivendo e caso, seja efetiva tem poder de se tornar escalável no sistema de saúde.

Critério 4: Os resultados têm possibilidade de expandir de forma significativa a fronteira do conhecimento na área e, portanto, de ter impacto científico relevante, com publicação de trabalhos com potencial de citação pela comunidade na literatura da área. A proposta em análise atende ao critério:

[ ] Muito [X] Bastante [ ] Razoavelmente [ ] Pouco [ ] Nada

Por favor, justifique a escolha acima (A força da justificativa será fator decisivo na análise da proposta pela FAPESP):

Sem comentários adicionais aos expostos no parecer inicial.

Critério 5: O projeto de pesquisa pode ser realizado pelo pesquisador responsável e sua equipe no prazo previsto na proposta. A proposta em análise atende ao critério:

[ ] Muito [X] Bastante [ ] Razoavelmente [ ] Pouco [ ] Nada

Por favor, justifique a escolha acima (A força da justificativa será fator decisivo na análise da proposta pela FAPESP):

Considero que a proponente tem os meios adequados em termos de infra-estrutura, apoio institucional e alunos envolvidos para dar conta da realização do estudo.

Critério 6: O Plano de Gestão de Dados descreve os tipos de dados produzidos pelo projeto,as formas de seu armazenamento, preservação e compartilhamento. A proposta em análise atende ao critério:

[ ] Muito [X] Bastante [ ] Razoavelmente [ ] Pouco [ ] Nada

Por favor, justifique a escolha acima (A força da justificativa será fator decisivo na análise da proposta pela FAPESP):

A proposta de gestão de dados melhorou significativamente em comparação à exposta anteriormente. Desafios de armazenamento de um conjunto grande de dados persiste, mas pode ser resolvido com suporte da própria universidade.

II. Sobre o HISTÓRICO ACADÊMICO do Pesquisador Responsável pelo Projeto

----

Critério 7: Os resultados científicos publicados pelo Pesquisador Responsável na área de pesquisa do projeto proposto têm impacto científico relevante. A proposta em análise atende ao critério:

[ ] Muito [X] Bastante [ ] Razoavelmente [ ] Pouco [ ] Nada

Por favor, justifique a escolha acima (A força da justificativa será fator decisivo na análise da proposta pela FAPESP):

Sem comentários adicionais aos expostos na avaliação inicial.

Critério 8: O pesquisador demonstra ter liderança científica ou, no caso de pesquisadores em início de carreira, seu potencial de liderança está evidenciado. A proposta em análise atende ao critério:

[ ] Muito [X] Bastante [ ] Razoavelmente [ ] Pouco [ ] Nada

Por favor, justifique a escolha acima (A força da justificativa será fator decisivo na análise da proposta pela FAPESP):

Sem comentários adicionais.

Critério 9: O pesquisador tem experiência na formação de pesquisadores compatível com o estágio da carreira e as condições institucionais. A proposta em análise atende ao critério:

[X] Muito [ ] Bastante [ ] Razoavelmente [ ] Pouco [ ] Nada

Por favor, justifique a escolha acima (A força da justificativa será fator decisivo na análise da proposta pela FAPESP):

Na nova súmula curricular fica mais evidente que a proponente está construindo uma trajetória consistente em termos da formação de recursos humanos e com liderança por meio de Grupo de Pesquisa.

Critério 10: O histórico acadêmico do Pesquisador demonstra experiência internacional em pesquisa após o doutoramento ou participação ativa em redes internacionais de colaboração em pesquisa. A proposta em análise atende ao critério:

[X] Muito [ ] Bastante [ ] Razoavelmente [ ] Pouco [ ] Nada

Por favor, justifique a escolha acima (A força da justificativa será fator decisivo na análise da proposta pela FAPESP):

Considero bastante promissor o vínculo com a Instituição NeuRA formalizado em janeiro de 2021 por meio de um Memorandum of understanding sob responsabilidade da candidata e do Prof. Stephen Lord. A inserção da candidata no

rede de pesquisa RAPID Physiotherapist Network, a
convite da pesquisadora da NeuRA Morag Taylor, do Falls, Balance and Injury Research Centre trará certamente a possibilidade de novos projetos de pesquisa e ampliará sua coloaboração internacional.

III. Sobre o ORÇAMENTO solicitado

Avaliação 1: Os equipamentos e materiais permanentes solicitados para a realização do projeto, face à infraestrutura existente na Instituição Sede, e à capacidade da equipe solicitante em utilizá-los, estão bem justificadas na proposta?

[X] Sim
[ ] Não

Opine, em cada caso, sobre a validade das justificativas apresentadas, considerando a necessidade para os objetivos da pesquisa e a eventual disponibilidade de similares na própria instituição. (O documento "Parque de Equipamentos", de apresentação obrigatória para análise, deve apresentar lista de equipamentos disponíveis na instituição).

Avaliação 2: Os itens de material de consumo solicitados para a realização do projeto têm sua necessidade justificada na proposta.

[X] Sim
[ ] Não

Opine, em cada caso, sobre a validade das justificativas apresentadas, considerando a necessidade para os objetivos da pesquisa.

Avaliação 3: Os Serviços de Terceiros solicitados para a realização do projeto têm sua necessidade justificada na proposta e são apenas de natureza técnica e eventual, conforme exigido pelas normas da FAPESP.

[X] Sim
[ ] Não

Opine, em cada caso, sobre a validade das justificativas apresentadas, considerando a necessidade para os objetivos da pesquisa.

A reformulação realizada está adequada.

Caso necessário ou oportuno, por favor, sugira valores alternativos para o orçamento. Observação Importante: devem ser excluídos do orçamento salários de qualquer natureza, serviços de terceiros que não de natureza técnica e eventual, obras civis, aquisição de publicações, viagens (exceto para pesquisa de campo), material e serviços administrativos. ORÇAMENTO SOLICITADO:

#	Rubrica	Qtd	Solicitado R$	Solicitado US$	Qtd	Recomendado R$	Recomendado US$
**1**	**Capital e Custeio**		**42.509,15**	**977,50**		**42.509,15**	**977,50**
2	Material Permanente		9.979,30	0,00		9.979,30	0,00
3	Material de Consumo		2.319,99	0,00		2.319,99	0,00
4	Despesas de Transporte		6.999,00	0,00		6.999,00	0,00
5	Serviços de Terceiros		500,00	850,00		500,00	850,00
6	Diárias		2.660,00	0,00		2.660,00	0,00
7	Reserva Técnica						
8	Reserva Técnica - Benefícios Complementares	1	16.000,00	0,00	1	16.000,00	0,00
9	Reserva Técnica - Custo de Infraestrutura Direta do Projeto		4.050,86	0,00		4.050,86	0,00
10	Provisão para Importação		0,00	127,50		0,00	127,50

#	Categoria	Mens.	R$	US$	Mens.	R$	US$
**11**	**Bolsas**		**14.740,80**	**0,00**		**29.481,60**	**0,00**
12	Treinamento Técnico	24	14.740,80	0,00	24	29.481,60	0,00
13	TT-1	0	0,00	0,00	0	0,00	0,00
14	TT-2	0	0,00	0,00	0	0,00	0,00
15	TT-3	24	29.481,60	0,00	24	29.481,60	0,00
16	TT-4	0	0,00	0,00	0	0,00	0,00
17	TT-4A	0	0,00	0,00	0	0,00	0,00

18	TT-5	0	0,00	0,00	0	0,00	0,00
19	Res. Téc. de Bolsas		0,00	0,00		0,00	0,00
**20**	**Total geral**		**57.249,95**	**977,50**		**71.990,75**	**977,50**

**Modalidade de Bolsa**	**Valores**
TT-1	R$ 439,60
TT-2	R$ 878,00
TT-3	R$ 1.228,40
TT-4	R$ 3.104,80
TT-4A	R$ 5.087,20
TT-5	R$ 7.372,40

IV. APRECIAÇÃO GERAL DA PROPOSTA (AGP)

Pontos Fortes (em particular, aponte qual o aspecto mais original ou inovador do projeto de pesquisa proposto)

----

a) Sobre o Projeto de Pesquisa, conforme indicado no item I:

[ ] Projeto com objetivos mal definidos, excessivos ou incongruentes.
[ ] Projeto com objetivos excessivamente limitados.
[ ] Projeto pouco original.
[ ] Desafios de pesquisa mal formulados.
[ ] Contribuição pouco significativa para a área do conhecimento
[ ] Metodologia inadequada.
[ ] Viabilidade de execução questionável.
[ ] Prazo inadequado.
[ ] Plano de Gestão de Dados inadequado e/ou insuficiente

----

b) Sobre o Pesquisador Responsável, conforme indicado no item II:

[ ] Experiência insuficiente na área de pesquisa em que se insere o projeto, podendo comprometer sua viabilidade.
[ ] Produção científica ou tecnológica que não atesta significativo rendimento da atividade de pesquisa.
[ ] Experiência insuficiente na formação de pesquisadores
[ ] Pouca experiência e inserção internacional.

----

c) Sobre o Orçamento proposto, conforme indicado no item III

[ ] Custo excessivo frente à contribuição científica ou tecnológica esperada ou à probabilidade de sucesso do projeto.
[ ] Equipamentos e Materiais Permanentes insuficientemente justificados.
[ ] Itens de Material de Consumo insuficientemente justificados.
[ ] Serviços de Terceiros insuficientemente justificados

----

d) Pontos Fracos (Outros - justificar):

Conclusão

Considero que a proponente fez reformulações no projeto que tornaram a proposta mais robusta. Trata-se de um estudo de natureza complexa e com inúmeros desafios. Os resultados podem ajudar a delinear uma estratégia de prevenção de quedas alinhada aos pressupostos do envelhecimento saudável, com aplicabilidade para o sistema de saúde e com contribuição para o avanço científico na área.

================================

PARECER DAS COORDENAÇÕES

Os questionamentos colocados pela assessoria foram abordados de forma detalhada na resposta à diligência e incorporados ao projeto. Com isto, a proposta pode ser recomendada. Recomenda-se que estes aspectos sejam abordados em detalhe quando do primeiro relatório.

Frases para Termo de Outorga

Não há frases associadas.

Orçamento Detalhado - Quadros Resumos

Material Permanente - Nacional

Item	Descrição	Solicitado			Despacho		
		Qtd	Valor Unitário (R$)	Valor Total(R$)	Qtd	Valor Unitário (R$)	Valor Total(R$)
4	Cronômetro profissional Kalenji	3	99,90	299,70	0	0,00	0,00
5	Esfigmomanômetro digital pulso	20	126,68	2.533,60	0	0,00	0,00
7	Impressora Epson EcoTank (L4160) Multifuncional	1	1.399,00	1.399,00	1	1.399,00	1.399,00
8	Kit arco de luz e tripé para celular	3	100,00	300,00	0	0,00	0,00
9	Notebook Acer Aspire 3 Intel Core i5	1	2.900,00	2.900,00	1	2.900,00	2.900,00
11	Tablet	3	849,00	2.547,00	3	849,00	2.547,00
Total				9.979,30			6.846,00

Material Permanente - Importado

Nenhum benefício encontrado.

Despesas de Transporte - Nacional

Item	Descrição	Solicitado			Despacho		
		Qtd	Valor Unitário (R$)	Valor Total(R$)	Qtd	Valor Unitário (R$)	Valor Total(R$)
2	ida e volta Sydney (Australia)-São Carlos (Brasil)-Sydney (A...	1	6.999,00	6.999,00	1	6.999,00	6.999,00
Total				6.999,00			6.999,00

Despesas de Transporte - Importado

Nenhum benefício encontrado.

Diárias - Nacional

Item	Descrição	Solicitado			Despacho		
		Qtd	Valor Unitário (R$)	Valor Total(R$)	Qtd	Valor Unitário (R$)	Valor Total(R$)
1	Diárias em São Carlos para pesquisador visitante	7	380,00	2.660,00	7	380,00	2.660,00
Total				2.660,00			2.660,00

Diárias - Importado

Nenhum benefício encontrado.

Material de Consumo - Nacional

Item	Descrição	Solicitado	Despacho
		Valor Total(R$)	Valor Total(R$)
3	Faixas elásticas Theraband de diferentes intensidades	289,32	289,32
6	Halteres de diferentes pesos (0,5 a 2 kg)	358,04	358,04
9	Materiais para divulgação (panfletos, cartões de visita, car...	500,00	500,00
10	Papel sulfite A4	91,96	0,00
11	Toner para impressora	244,90	0,00
12	Tornozeleiras de diferentes pesos (1 a 5 kg)	835,77	835,77
13	Cronômetro profissional Kalenji	-	299,70
14	Esfigmomanômetro digital pulso	-	2.533,60
15	Kit arco de luz e tripé para celular	-	300,00
Total		2.319,99	5.116,43

Material de Consumo - Importado

Nenhum benefício encontrado.

Serviços de Terceiros - Nacional

Item	Descrição	Solicitado			Despacho		
		Qtd	Valor Unitário (R$)	Valor Total(R$)	Qtd	Valor Unitário (R$)	Valor Total(R$)
4	Serviço de correio para entrega de materiais	1	500,00	500,00	1	500,00	500,00
Total				500,00			500,00

Serviços de Terceiros - Importado

Item	Descrição	Solicitado				Despacho			
		MO* / Tx. Conv.(US$)	Qtd	Valor Unitário	Valor Total(US$)	MO* / Tx. Conv.(US$)	Qtd	Valor Unitário	Valor Total(US$)
3	Revisão básica de artigo para o inglês - 3000 palavras AJE	US$/1,0000000	5	75,00	375,00	US$/1,0000000	5	75,00	375,00
5	Tradução de artigo para o inglês - 3000 palavras AJE	US$/1,0000000	1	475,00	475,00	US$/1,0000000	1	475,00	475,00
Total					850,00				850,00

* MO = Moeda de Origem

Reserva Técnica - Benefícios Complementares

	Nome	Papel	Valor	Vigência
**Beneficiados**		Pesquisador Responsável	32.000,00	01/02/2022 a 31/01/2024

**Moeda**	R$
**Valor Unitário** (anual)	16.000,00
**Data de Referência**	29/11/2021
**Valor do Benefício Complementar**	32.000,00

Reserva Técnica - Custo de Infraestrutura Direta do Projeto - Solicitado

**Percentual para Reserva Técnica (País)**	15,00 %
**Percentual para Reserva Técnica (Exterior)**	15,00 %
**Dólar FAPESP**	5,35
**Valor da Reserva Técnica (R$)**	4.050,86
**Valor da Reserva Técnica (US$)**	0,00

Reserva Técnica - Custo de Infraestrutura Direta do Projeto - Despacho

**Percentual para Reserva Técnica (País)**	15,00 %
**Percentual para Reserva Técnica (Exterior)**	15,00 %
**Dólar FAPESP**	5,75
**Valor da Reserva Técnica (R$)**	4.051,33
**Valor da Reserva Técnica (US$)**	0,00

Provisão para Importação - Solicitado

**Percentual para Provisão para Importação (Exterior)**	15,00 %
**Valor da Provisão para Importação (US$)**	127,50

Provisão para Importação - Despacho

**Percentual para Provisão para**	15,00 %

**Importação**	
**Valor da Provisão para Importação (US$)**	127,50

Orçamento Detalhado - Itens de despesa

Material Permanente - Nacional

**Origem**	Brasil
**Quantidade**	1
**Descrição**	Notebook Acer Aspire 3 Intel Core i5
**Fabricado no Brasil**	Sim
**Moeda de Origem**	R$
**Valor Unitário**	2.900,00
**Valor Total**	2.900,00
**Justificativa**	A aquisição do microcomputador auxiliará no armazenamento e processamento dos dados coletados. A impressora auxiliará na impressão de dados, relatórios, fichas e avaliação.

Material Permanente - Nacional

**Origem**	Brasil
**Quantidade**	3
**Descrição**	Tablet
**Fabricado no Brasil**	Sim
**Moeda de Origem**	R$
**Valor Unitário**	849,00
**Valor Total**	2.547,00
**Justificativa**	Materiais permanentes para avaliação, indispensáveis para sua padronização.

Material Permanente - Nacional

**Origem**	Brasil
**Quantidade**	1
**Descrição**	Impressora Epson EcoTank (L4160) Multifuncional
**Fabricado no Brasil**	Sim
**Moeda de Origem**	R$
**Valor Unitário**	1.399,00
**Valor Total**	1.399,00
**Justificativa**	A aquisição do microcomputador auxiliará no armazenamento e processamento dos dados coletados. A impressora auxiliará na impressão de dados, relatórios, fichas e avaliação.

Material Permanente - Nacional

**Origem**	Brasil
**Quantidade**	0
**Descrição**	Cronômetro profissional Kalenji
**Fabricado no Brasil**	Sim
**Moeda de Origem**	R$
**Valor Unitário**	0,00
**Valor Total**	0,00
**Justificativa**	Materiais permanentes para avaliação, indispensáveis para sua padronização.

Material Permanente - Nacional

**Origem**	Brasil
**Quantidade**	0
**Descrição**	Kit arco de luz e tripé para celular
**Fabricado no Brasil**	Sim
**Moeda de Origem**	R$

**Valor Unitário**	0,00
**Valor Total**	0,00
**Justificativa**	Material para gravação de vídeo, a ser utilizada na intervenção remota.

Material Permanente - Nacional

**Origem**	Brasil
**Quantidade**	0
**Descrição**	Esfigmomanômetro digital pulso
**Fabricado no Brasil**	Sim
**Moeda de Origem**	R$
**Valor Unitário**	0,00
**Valor Total**	0,00
**Justificativa**	Material permanente para segurança dos voluntários do Grupo Intervenção, a ser utilizada antes e depois das sessões de exercício físico domiciliar pelos próprios voluntários.

Despesas de Transporte - Nacional

**Origem**	Brasil
**Quantidade**	1
**Descrição**	ida e volta Sydney (Australia)-São Carlos (Brasil)-Sydney (Australia) para professor visitante
**Valor Unitário**	6.999,00
**Valor Total**	6.999,00
**Justificativa**	Pretende-se realizar solicitações de Auxílio Pesquisador Visitante durante o período de vigência do projeto, caso este seja aprovado e financiado. O Auxílio Pesquisador Visitante será importante para fortalecer a parceria internacional entre a equipe de pesquisa brasileira e a Neuroscience Research Australia (NeuRA), da University of New South Wales, liderada pelo Prof. Dr. Stephen Lord. Trata-se de uma instituição líder em pesquisa sobre quedas e controle postural em diferentes populacionais, incluindo a idosa. Ressalta-se que no final de 2016 a pesquisadora responsável realizou visita técnica na NeuRA e em 2017 foi publicado um artigo, fruto da parceria, no American Journal of Physical Medicine & Rehabilitation. O Auxílio Pesquisador Visitante será primordial para o estreitamento do vínculo e a troca de experiências e expertises, por meio de vindas de pesquisa visitante colaborador e visitas técnicas

Diárias - Nacional

**Quantidade**	7	
**Descrição**	Diárias em São Carlos para pesquisador visitante	
**Local**	Brasil	
	**Estado**	**Cidade**
	São Paulo	São Carlos
**Pernoite**	Sim	
**Moeda**	R$	
**Valor Unitário**	380,00	
**Valor Total**	2.660,00	
**Justificativa**	Pretende-se realizar solicitações de Auxílio Pesquisador Visitante durante o período de vigência do projeto, caso este seja aprovado e financiado. O Auxílio Pesquisador Visitante será importante para fortalecer a parceria internacional entre a equipe de pesquisa brasileira e a Neuroscience Research Australia (NeuRA), da University of New South Wales, liderada pelo Prof. Dr. Stephen Lord. Trata-se de uma instituição líder em pesquisa sobre quedas e controle postural em diferentes populacionais, incluindo a idosa. Ressalta-se que no final de 2016 a pesquisadora responsável realizou visita técnica na NeuRA e em 2017 foi publicado um artigo, fruto da parceria, no American Journal of Physical Medicine & Rehabilitation. O Auxílio Pesquisador Visitante será primordial para o estreitamento do vínculo e a troca de experiências e expertises, por meio de vindas de pesquisa visitante colaborador e visitas técnicas.	

Material de Consumo - Nacional

**Origem**	Brasil
**Descrição**	Esfigmomanômetro digital pulso
**Valor Unitário**	2.533,60
**Valor Total**	2.533,60
**Justificativa**	Material permanente para segurança dos voluntários do Grupo Intervenção, a ser

	utilizada antes e depois das sessões de exercício físico domiciliar pelos próprios voluntários.

Material de Consumo - Nacional

**Origem**	Brasil
**Descrição**	Tornozeleiras de diferentes pesos (1 a 5 kg)
**Valor Unitário**	835,77
**Valor Total**	835,77
**Justificativa**	Materiais de consumo para intervenção do estudo e protocolo de exercício físico domiciliar.

Material de Consumo - Nacional

**Origem**	Brasil
**Descrição**	Materiais para divulgação (panfletos, cartões de visita, cartazes)
**Valor Unitário**	500,00
**Valor Total**	500,00
**Justificativa**	Material para divulgação e recrutamento.

Material de Consumo - Nacional

**Origem**	Brasil
**Descrição**	Halteres de diferentes pesos (0,5 a 2 kg)
**Valor Unitário**	358,04
**Valor Total**	358,04
**Justificativa**	Materiais de consumo para intervenção do estudo e protocolo de exercício físico domiciliar.

Material de Consumo - Nacional

**Origem**	Brasil
**Descrição**	Kit arco de luz e tripé para celular
**Valor Unitário**	300,00
**Valor Total**	300,00
**Justificativa**	Material para gravação de vídeo, a ser utilizada na intervenção remota.

Material de Consumo - Nacional

**Origem**	Brasil
**Descrição**	Cronômetro profissional Kalenji
**Valor Unitário**	299,70
**Valor Total**	299,70
**Justificativa**	Materiais permanentes para avaliação, indispensáveis para sua padronização.

Material de Consumo - Nacional

**Origem**	Brasil
**Descrição**	Faixas elásticas Theraband de diferentes intensidades
**Valor Unitário**	289,32
**Valor Total**	289,32
**Justificativa**	Materiais de consumo para intervenção do estudo e protocolo de exercício físico domiciliar.

Material de Consumo - Nacional

**Origem**	Brasil
**Descrição**	Papel sulfite A4
**Valor Unitário**	0,00
**Valor Total**	0,00
**Justificativa**	Tais materiais auxiliarão na impressão de dados, relatórios, fichas, avaliação e intervenção (cartilha, recomendações).

Material de Consumo - Nacional

**Origem**	Brasil
**Descrição**	Toner para impressora

**Valor Unitário**	0,00
**Valor Total**	0,00
**Justificativa**	Tais materiais auxiliarão na impressão de dados, relatórios, fichas, avaliação e intervenção (cartilha, recomendações).

Serviços de Terceiros - Importado

**Origem**	Exterior
**Quantidade**	1
**Descrição**	Tradução de artigo para o inglês - 3000 palavras AJE
**Moeda de Origem**	US$
**Valor Unitário**	475,00
**Taxa de Câmbio (US$)**	1,0000000
**Valor Total**	475,00
**Justificativa**	Serviços de revisão ortográfica e tradução de artigos para publicações em periódicos de caráter internacional.

Serviços de Terceiros - Importado

**Origem**	Exterior
**Quantidade**	5
**Descrição**	Revisão básica de artigo para o inglês - 3000 palavras AJE
**Moeda de Origem**	US$
**Valor Unitário**	75,00
**Taxa de Câmbio (US$)**	1,0000000
**Valor Total**	375,00
**Justificativa**	Serviços de revisão ortográfica e tradução de artigos para publicações em periódicos de caráter internacional.

Serviços de Terceiros - Nacional

**Origem**	Brasil
**Quantidade**	1
**Descrição**	Serviço de correio para entrega de materiais
**Valor Unitário**	500,00
**Valor Total**	500,00
**Justificativa**	Recurso para entrega de materiais de intervenção, como cartilhas e recomendações.

Reserva Técnica - Benefícios Complementares

	Nome	Papel	Valor	Vigência
**Beneficiados**		Pesquisador Responsável	32.000,00	01/02/2022 a 31/01/2024

**Moeda**	R$
**Valor Unitário** (anual)	16.000,00
**Data de Referência**	29/11/2021
**Valor do Benefício Complementar**	32.000,00

Reserva Técnica - Custo de Infraestrutura Direta do Projeto

**Percentual para Reserva Técnica (País)**	15,00 %
**Percentual para Reserva Técnica (Exterior)**	15,00 %
**Dólar FAPESP**	5,75
**Valor da Reserva Técnica (R$)**	4.051,33
**Valor da Reserva Técnica (US$)**	0,00

Provisão para Importação

**Percentual para Provisão para Importação**	15,00 %
**Valor da Provisão para Importação (US$)**	127,50

FAPESP SAGe

VISUALIZAÇÃO DE DESPACHO

**Processo**	2021/01372-5
**Linha de Fomento**	Programas Regulares / Auxílios a Pesquisa / Projeto de Pesquisa / Projeto de Pesquisa - Regular - Fluxo Contínuo
**Situação**	Em Contratação
**Vigência**	01/07/2021 a 30/06/2023
**Beneficiário**	Karina Gramani Say
**Responsável**	Karina Gramani Say
**Vínculo Institucional do Processo**	Centro de Ciências Biológicas e da Saúde/CCBS/UFSCAR
**Título**	Aspectos funcionais e Avaliação econômica de saúde da gestão de casos em um programa de prevenção de quedas para idosos caidores da comunidade: estudo clínico controlado randomizado

Folha de Despacho para Proposta Inicial - Projeto de Pesquisa - Regular

Resultado

Concedido

Datas do Despacho

**Emitido em :**	25/05/2021

Carregando...

Carregando...

Carregando...

Dados de Execução

**Data Início**	01/07/2021
**Duração**	24 mês(es)
**Data Término**	30/06/2023
**Área de alocação de recursos**	Saúde
**Relatório Científico (Quantidade)**	2
**Relatório Científico (Datas)**	30/06/2022<br>30/07/2023
**Prestação de Contas (Quantidade)**	2
**Prestação de Contas (Datas)**	30/06/2022<br>30/07/2023
**Categoria de Pesquisa**	B/T/PP

Observações / Transcrições / Frases

Observações ao Responsável

Comunicamos que sua solicitação de auxílio à pesquisa, constante do processo acima referido, foi analisada pela assessoria da FAPESP, tendo sido aprovada.

Cabe-nos informar que alguns dos itens orçamentários solicitados podem não ter sido aprovados, ou aprovados com valores inferiores. Por favor, aguarde o email com as instruções para confirmação de interesse pela concessão.

A utilização da concessão deve seguir o Manual de Instruções para Uso dos Recursos e Prestação de Contas de Auxílios e de Reserva Técnica.

Para conhecimento do conteúdo do despacho, por favor, acesse o Sistema SAGe (www.fapesp.br/sage), selecionando o item do menu Meus Processos>>Número do Processo e, em Mais Informações, a opção Despacho.

Por favor, para qualquer consulta ou comunicação sobre esta correspondência, use exclusivamente os serviços do "Converse com a FAPESP" em www.fapesp.br/converse.

Atenciosamente,

Luiz Eugênio A. M. Mello
Diretor Científico

OBSERVAÇÃO:
1. Foi concedida cota de bolsa orçamentária, conforme discriminado no Termo de Outorga, podendo haver dedução de bolsa anterior quando a regra se aplicar.
O candidato selecionado somente poderá iniciar suas atividades no projeto após a aprovação de seu cadastro, que deve ser submetido via SAGe, conforme Instrução Normativa específica para cada modalidade, constante do site da FAPESP em www.fapesp.br/bolsas/bolsasconcedidasemauxilios. Não serão feitos pagamentos ou reembolsos referentes a bolsistas fora do respectivo processo SAGe.
Possíveis renovações da referida cota de bolsa deverão ser solicitadas no Auxilio, por meio de solicitação de alteração da concessão inicial, no momento da entrega do Relatório Científico. A vigência da bolsa não poderá ultrapassar a vigência do auxílio.

Frases para o Responsável

Não há frases associadas.

Transcrição de Parecer para o Responsável

ANÁLISE DA PROPOSTA. A análise de propostas submetidas na modalidade Auxílio à Pesquisa Regular compreende a Avaliação do Projeto de Pesquisa, do Histórico Acadêmico do Pesquisador Responsável pelo Projeto e do Orçamento. Este formulário é composto por três seções referentes a cada uma das partes, tendo ao final uma Apreciação Geral do Projeto de Pesquisa (AGP).

I. Sobre o PROJETO DE PESQUISA proposto

Critério 1: Projeto de pesquisa original, competitivo internacionalmente e bem apresentado. A proposta em análise atende ao critério:

[ ] Muito [X] Bastante [ ] Razoavelmente [ ] Pouco [ ] Nada

Por favor, justifique a escolha acima (A força da justificativa será fator decisivo na análise da proposta pela FAPESP):

A preocupação com a população idosa é um fenômeno mundial e necessita de maior compreensão principalmente em países não desenvolvidos, como o Brasil. A realidade nacional, por inúmeros aspectos, se diferencia da situação em outros países. sendo assim, investigar a situação local torna-se fundamental para gerar subsídios na gestão de saúde pública.

Critério 2: Os desafios de pesquisa estão adequadamente formulados e situados frente ao estado da arte e à literatura existente. A proposta em análise atende ao critério:

[X] Muito [ ] Bastante [ ] Razoavelmente [ ] Pouco [ ] Nada

Por favor, justifique a escolha acima (A força da justificativa será fator decisivo na análise da proposta pela FAPESP):

A proposta está bem elaborada, considerando os conhecimentos já adquiridos até o momento e propõe investigar a viabilidade de uma proposta promissora de intervenção, com expectativa de ótimos resultados e baixo custo.

Critério 3: A metodologia é adequada e está bem justificada e suficientemente detalhada. A proposta em análise atende ao critério:

[X] Muito [ ] Bastante [ ] Razoavelmente [ ] Pouco [ ] Nada

Por favor, justifique a escolha acima (A força da justificativa será fator decisivo na análise da proposta pela FAPESP):

O delineamento metodológico está atendendo todos aspectos necessários para responder aos objetivos elencados, além de ter potencial para ter um grau de evidência científica muito satisfatório.

Critério 4: Os resultados têm possibilidade de expandir de forma significativa a fronteira do conhecimento na área e, portanto, de ter impacto científico relevante, com publicação de trabalhos com potencial de citação pela comunidade na literatura da área. A proposta em análise atende ao critério:

[X] Muito [ ] Bastante [ ] Razoavelmente [ ] Pouco [ ] Nada

Por favor, justifique a escolha acima (A força da justificativa será fator decisivo na análise da proposta pela FAPESP):

Por se tratar de uma temática muito explorada mundialmente , que carece de mais dados referente a situação local, em país não desenvolvido, o potencial para publicação científica relevante em revistas de gerontologia é bastante expressivo.

Critério 5: O projeto de pesquisa pode ser realizado pelo pesquisador responsável e sua equipe no prazo previsto na proposta. A proposta em análise atende ao critério:

[X] Muito [ ] Bastante [ ] Razoavelmente [ ] Pouco [ ] Nada

Por favor, justifique a escolha acima (A força da justificativa será fator decisivo na análise da proposta pela FAPESP):

A equipe elencada parece ser bastante robusta para dar andamento no projeto. O cronograma está bem viável, inclusive tomaram o cuidado de prever medidas necessárias em função da Pandemia COVID-19.

Critério 6: O Plano de Gestão de Dados descreve os tipos de dados produzidos pelo projeto,as formas de seu armazenamento, preservação e compartilhamento. A proposta em análise atende ao critério:

[ ] Muito [ ] Bastante [X] Razoavelmente [ ] Pouco [ ] Nada

Por favor, justifique a escolha acima (A força da justificativa será fator decisivo na análise da proposta pela FAPESP):

O aspecto que precisa ser melhorado na proposta é o "Plano de Gestão de Dados", pois o mesmo não está detalhado em como será realizado o armazenamento, preservação e compartilhamento dos dados obtidos. Foi feito menção quase que exclusivamente sobre quais dados serão obtidos e informa que o compartilhamento será por meio de artigos científicos. Não ficou claro como e quais condições os dados obtidos poderão ser buscados e acessados por pesquisadores.

II. Sobre o HISTÓRICO ACADÊMICO do Pesquisador Responsável pelo Projeto

----

Critério 7: Os resultados científicos publicados pelo Pesquisador Responsável na área de pesquisa do projeto proposto têm impacto científico relevante. A proposta em análise atende ao critério:

[ ] Muito [X] Bastante [ ] Razoavelmente [ ] Pouco [ ] Nada

Por favor, justifique a escolha acima (A força da justificativa será fator decisivo na análise da proposta pela FAPESP):

A pesquisadora responsável vem aprimorando sua atuação na área de gerontologia, com publicações relevantes em vários aspectos da área, que por essência é multidisciplinar.

Critério 8: O pesquisador demonstra ter liderança científica ou, no caso de pesquisadores em início de carreira, seu potencial de liderança está evidenciado. A proposta em análise atende ao critério:

[ ] Muito [X] Bastante [ ] Razoavelmente [ ] Pouco [ ] Nada

Por favor, justifique a escolha acima (A força da justificativa será fator decisivo na análise da proposta pela FAPESP):

A gerontologia, como área de formação universitária e de conhecimento é bastante recente no Brasil. A pesquisadora, considerada em início de carreira nesta área, demostra potencial em liderança de projetos desta natureza.

Critério 9: O pesquisador tem experiência na formação de pesquisadores compatível com o estágio da carreira e as condições institucionais. A proposta em análise atende ao critério:

[ ] Muito [X] Bastante [ ] Razoavelmente [ ] Pouco [ ] Nada

Por favor, justifique a escolha acima (A força da justificativa será fator decisivo na análise da proposta pela FAPESP):

A pesquisadora tem regularidade na orientação e coorientação de pós-graduandos, além de envolvimento com iniciação científica.

Critério 10: O histórico acadêmico do Pesquisador demonstra experiência internacional em pesquisa após o doutoramento ou participação ativa em redes internacionais de colaboração em pesquisa. A proposta em análise atende ao critério:

[ ] Muito [X] Bastante [ ] Razoavelmente [ ] Pouco [ ] Nada

Por favor, justifique a escolha acima (A força da justificativa será fator decisivo na análise da proposta pela FAPESP):

Na proposta foi inserido pareceria com pesquisador internacional.

III. Sobre o ORÇAMENTO solicitado

Avaliação 1: Os equipamentos e materiais permanentes solicitados para a realização do projeto, face à infraestrutura existente na Instituição Sede, e à capacidade da equipe solicitante em utilizá-los, estão bem justificadas na proposta?

[X] Sim
[ ] Não

Opine, em cada caso, sobre a validade das justificativas apresentadas, considerando a necessidade para os objetivos da pesquisa e a eventual disponibilidade de similares na própria instituição. (O documento "Parque de Equipamentos", de apresentação obrigatória para análise, deve apresentar lista de equipamentos disponíveis na instituição).

Os equipamentos, de baixo custo, foram elencados em quantidade satisfatória para o andamento da pesquisa.

Avaliação 2: Os itens de material de consumo solicitados para a realização do projeto têm sua necessidade justificada na proposta.

[X] Sim
[ ] Não

Opine, em cada caso, sobre a validade das justificativas apresentadas, considerando a necessidade para os objetivos da pesquisa.

Estão de acordo com a necessidade da proposta.

Avaliação 3: Os Serviços de Terceiros solicitados para a realização do projeto têm sua necessidade justificada na proposta e são apenas de natureza técnica e eventual, conforme exigido pelas normas da FAPESP.

[X] Sim
[ ] Não

Opine, em cada caso, sobre a validade das justificativas apresentadas, considerando a necessidade para os objetivos da pesquisa.

Não foi observado solicitação de serviços de terceiros.

Caso necessário ou oportuno, por favor, sugira valores alternativos para o orçamento. Observação Importante: devem ser excluídos do orçamento salários de qualquer natureza, serviços de terceiros que não de natureza técnica e eventual, obras civis, aquisição de publicações, viagens (exceto para pesquisa de campo), material e serviços administrativos. ORÇAMENTO SOLICITADO:

#	Rubrica	Qtd	Solicitado R$	Solicitado US$	Qtd	Recomendado R$	Recomendado US$
1	**Capital e Custeio**		**57.342,50**	**0,00**		**57.342,50**	**0,00**
2	Material Permanente		30.930,00	0,00		30.930,00	0,00
3	Material de Consumo		1.820,00	0,00		1.820,00	0,00
4	Despesas de Transporte		3.200,00	0,00		3.200,00	0,00
5	Reserva Técnica						
6	Reserva Técnica - Benefícios Complementares	1	16.000,00	0,00	1	16.000,00	0,00
7	Reserva Técnica - Custo de Infraestrutura Direta do Projeto		5.392,50	0,00		5.392,50	0,00
8	Provisão para Importação		0,00	0,00		0,00	0,00

#	Categoria	Mens.	R$	US$	Mens.	R$	US$
9	**Bolsas**		**7.370,40**	**0,00**		**14.740,80**	**0,00**
10	Treinamento Técnico	12	7.370,40	0,00	12	14.740,80	0,00
11	TT-1	0	0,00	0,00	0	0,00	0,00
12	TT-2	0	0,00	0,00	0	0,00	0,00
13	TT-3	12	14.740,80	0,00	12	14.740,80	0,00
14	TT-4	0	0,00	0,00	0	0,00	0,00
15	TT-4A	0	0,00	0,00	0	0,00	0,00
16	TT-5	0	0,00	0,00	0	0,00	0,00
17	Res. Téc. de Bolsas		0,00	0,00		0,00	0,00
18	**Total geral**		**64.712,90**	**0,00**		**72.083,30**	**0,00**

Modalidade de Bolsa	Valores
TT-1	R$ 439,60
TT-2	R$ 878,00
TT-3	R$ 1.228,40
TT-4	R$ 3.104,80

TT-4A	R$ 5.087,20
TT-5	R$ 7.372,40

IV. APRECIAÇÃO GERAL DA PROPOSTA (AGP)

Pontos Fortes (em particular, aponte qual o aspecto mais original ou inovador do projeto de pesquisa proposto)

A proposta tem como ponto forte ser uma proposta de intervenção e acompanhamento de idosos por meio de estratégias com grande potencial de eficácia e baixo custo, podendo gerar subsídios científicos para implementação em Política Nacional de saúde Pública para idosos.

----

a) Sobre o Projeto de Pesquisa, conforme indicado no item I:

[ ] Projeto com objetivos mal definidos, excessivos ou incongruentes.
[ ] Projeto com objetivos excessivamente limitados.
[ ] Projeto pouco original.
[ ] Desafios de pesquisa mal formulados.
[ ] Contribuição pouco significativa para a área do conhecimento
[ ] Metodologia inadequada.
[ ] Viabilidade de execução questionável.
[ ] Prazo inadequado.
[X] Plano de Gestão de Dados inadequado e/ou insuficiente

----

b) Sobre o Pesquisador Responsável, conforme indicado no item II:

[ ] Experiência insuficiente na área de pesquisa em que se insere o projeto, podendo comprometer sua viabilidade.
[ ] Produção científica ou tecnológica que não atesta significativo rendimento da atividade de pesquisa.
[ ] Experiência insuficiente na formação de pesquisadores
[ ] Pouca experiência e inserção internacional.

----

c) Sobre o Orçamento proposto, conforme indicado no item III

[ ] Custo excessivo frente à contribuição científica ou tecnológica esperada ou à probabilidade de sucesso do projeto.
[ ] Equipamentos e Materiais Permanentes insuficientemente justificados.
[ ] Itens de Material de Consumo insuficientemente justificados.
[ ] Serviços de Terceiros insuficientemente justificados

----

d) Pontos Fracos (Outros - justificar):

Conclusão

Após maior detalhamento do Plano de Gestão de dados, considero ser viável aprovar o referido Projeto.

================================

PARECER DAS COORDENAÇÕES

A proposta foi bem avaliada nos seus principais aspectos e poderá proporcionar contribuição científica relevante. Foram abertas diligências para correção no número de horas semanais de dedicação e do plano de gestão de dados.

Frases para Termo de Outorga

Não há frases associadas.

Carregando...
